# Opportunities and Challenges of Kava in Lung Cancer Prevention

**DOI:** 10.3390/ijms24119539

**Published:** 2023-05-31

**Authors:** Breanne Freeman, Jessica Mamallapalli, Tengfei Bian, Kayleigh Ballas, Allison Lynch, Alexander Scala, Zhiguang Huo, Kristianna M. Fredenburg, Adriaan W. Bruijnzeel, Carolyn J. Baglole, Junxuan Lu, Ramzi G. Salloum, John Malaty, Chengguo Xing

**Affiliations:** 1 Department of Medicinal Chemistry and Center for Natural Products, Drug Discovery and Development, College of Pharmacy, University of Florida, Gainesville, FL 32610, USA; b.freeman@ufl.edu (B.F.); jmamallapalli@ufl.edu (J.M.); tbian@ufl.edu (T.B.); kayleigh.ballas@ufl.edu (K.B.); lyncha1@ufl.edu (A.L.); a.scala@ufl.edu (A.S.); 2 Department of Biostatistics, College of Public Health & Health Professions, College of Medicine, University of Florida, Gainesville, FL 32610, USA; zhuo@ufl.edu; 3 Department of Pathology, Immunology and Laboratory Medicine, College of Medicine, University of Florida, Gainesville, FL 32610, USA; kfredenburg@ufl.edu; 4 Department of Psychiatry, College of Medicine, University of Florida, Gainesville, FL 32610, USA; awbruijn@ufl.edu; 5 Department of Medicine, McGill University, Montreal, QC H3A 0G4, Canada; carolyn.baglole@mcgill.ca; 6 Department of Pharmacology, PennState Cancer Institute, Penn State University College of Medicine, Hershey, PA 17033, USA; junxuanlu@pennstatehealth.psu.edu; 7 Department of Health Outcome & Biomedical Informatics, College of Medicine, University of Florida, Gainesville, FL 32610, USA; rsalloum@ufl.edu; 8 Department of Community Health & Family Medicine, College of Medicine, University of Florida, Gainesville, FL 32610, USA; malaty@ufl.edu

**Keywords:** lung cancer prevention, kava, tobacco smoke, lung carcinogenesis, risk factors, mechanism-based non-invasive biomarkers

## Abstract

Lung cancer is the leading cause of cancer-related deaths due to its high incidence, late diagnosis, and limited success in clinical treatment. Prevention therefore is critical to help improve lung cancer management. Although tobacco control and tobacco cessation are effective strategies for lung cancer prevention, the numbers of current and former smokers in the USA and globally are not expected to decrease significantly in the near future. Chemoprevention and interception are needed to help high-risk individuals reduce their lung cancer risk or delay lung cancer development. This article will review the epidemiological data, pre-clinical animal data, and limited clinical data that support the potential of kava in reducing human lung cancer risk via its holistic polypharmacological effects. To facilitate its future clinical translation, advanced knowledge is needed with respect to its mechanisms of action and the development of mechanism-based non-invasive biomarkers in addition to safety and efficacy in more clinically relevant animal models.

## 1. Introduction—Urgency of Lung Cancer Prevention

Lung cancer is the second most commonly diagnosed non-dermatological malignancy among both men and women globally, following prostate cancer and breast cancer, respectively [1,2]. At the same time, lung cancer has been the leading cause of cancer deaths for decades due to its relatively late diagnosis, poor treatment outcome, and high prevalence. There were about 2.2 million new lung cancer cases with 1.8 million deaths worldwide in 2020, representing approximately 11.4% of all cancers diagnosed and 18.0% of cancer caused deaths [1]. The average age for patients with lung cancer at time of diagnosis is roughly 70 years old. The 5-year survival rate of lung cancer patients has been increasing over the past four decades with improvements in early diagnosis and new treatment options, such as molecular-targeted therapies and immunotherapies; however, this rate is still very low—barely surpassing 22% in the USA in 2022 [2].

Based on clinical histopathology, primary lung cancer is typically divided into two main types: small cell lung cancer (SCLC) and non-small-cell lung cancer (NSCLC) [3]. SCLC accounts for about 15% of lung cancer cases with more than 90% of patients being elderly current or former heavy smokers [4]. NSCLC accounts for about 85% of lung cancer cases with three main subtypes, namely adenocarcinoma (ADC), squamous cell carcinoma (SCC), and large cell carcinoma (LCC). The incidence rates of SCLC, ADC, SCC, and LCC were 6.0, 17.9, 13.3, and 3.1/100,000 person-years, respectively [5]. The average 5-year survival rates for patients with SCLC, ADC, SCC, and LCC were 7.2%, 26.2%, 21.3%, and 21.1% in 2010, respectively [5]. Therefore, relative to NSCLC, SCLC has an exceptionally high mortality rate and median survival ranges between 7–10 months [6]. Although patients with NSCLC have a better survival rate than those with SCLC, the 5-year survival rate is much lower than other common cancers, such as prostate and breast cancers [1,2].

Based on clinical stage, lung cancer can be divided into four categories: localized, regional, distant metastatic, and un-staged. The average percentage of localized, regional, distant, and un-staged lung cancers at diagnosis is 18.8%, 23.7%, 47.7%, and 10.0%, respectively. Therefore, the majority of lung cancers are diagnosed at the distant metastatic stage [5]. Patients with localized stage lung cancer have the highest 5-year survival rate (50.3%), followed by regional stage lung cancer (22.2%), and then distant stage (2.9%) [5].

Lung cancer was a rare disease before the prevalent use of tobacco products. Historically lung cancer incidence rates have been higher among men than women because more men start smoking earlier and smoke at higher rates [7]. Female patients with lung cancer also have a better 5-year survival rate than male patients, potentially due to the lower level of exposure to tobacco products. The incidence rate of male patients with lung cancer in some countries shows a decreasing trend, while the rate for female patients shows an increasing trend in the past decades, likely due to progress in tobacco control overall, but increased prevalence of tobacco use among female populations [8]. The trends in lung cancer rates between developed countries and developing countries are also different [9]. In developed countries, lung cancer incidence and mortality rates are generally declining, again likely due to the reduced prevalence of tobacco use. In contrast, lung cancer incidence and mortality rates in developing countries are increasing, which is likely to continue, due to endemic use of tobacco products in these countries. This change is particularly obvious in Africa where lung cancer has been a rare disease even in recent decades due to limited tobacco exposure. The historical and epidemiological data provide compelling and unequivocal evidence that tobacco control should be promoted and implemented globally to improve the management of lung cancer and many other health conditions.

As previously mentioned, patients with localized stage lung cancer have the highest 5-year survival rate (50.3%) while the 5-year survival rate for patients with distant metastasis is only 2.9% [7]. Detection of lung cancer at an earlier stage therefore can greatly improve lung cancer management [10,11]. Early detection could be pursued with different approaches, such as the use of diagnostic imaging techniques among high-risk individuals. The National Lung Screening Trial (NLST) demonstrated a 20% reduction in mortality with low-dose CT (LDCT) screening [12]. However, there are some concerns about the limitations of this radiological screening tool, including the high-false positive rate, the potential for overdiagnosis, and the side effects from the radiation exposure [13]. Different biosources from liquid biopsy, including cell free circulating tumor DNA (ctDNA) [14], circulating tumor cells (CTCs) [15], exosomes [16] and tumor-educated platelets (TEPs) [17], have also been investigated for their potential role in lung cancer early diagnosis. So far, the ideal biosource and molecular biomarker for use in clinical diagnostics of lung malignancies have not yet been defined [18].

Due to the limited success in lung cancer early detection and clinical treatment, risk reduction or prevention is of paramount importance to decrease lung cancer burden. Given that 80–90% of lung cancers are associated with tobacco exposure, more stringent policies and regulations on tobacco production, sale, and use should be developed and implemented globally because reducing tobacco exposure would be the most effective strategy to reducing lung cancer incidence and burden from its root cause. Tobacco cessation should be highly promoted and more effectively implemented as well [19]. These strategies should also help reduce many other health conditions caused or worsened by tobacco exposure. However, because of the addictive nature of nicotine in tobacco products [20] and many other reasons that are beyond the scope of this review, reducing tobacco exposure has had limited success. Furthermore, there is little indication that the number of current and former smokers will decrease significantly in the near future [21]. Hence, preventive agents need to be developed for high-risk populations to reduce their risk of developing lung cancer. This is potentially feasible due to the latent nature of lung carcinogenesis, which requires decades for it to evolve into the clinically detectable stage [22].

## 2. Different Risk Factors Contributing to Lung Carcinogenesis via Multiple Mechanisms

**Figure 1:**
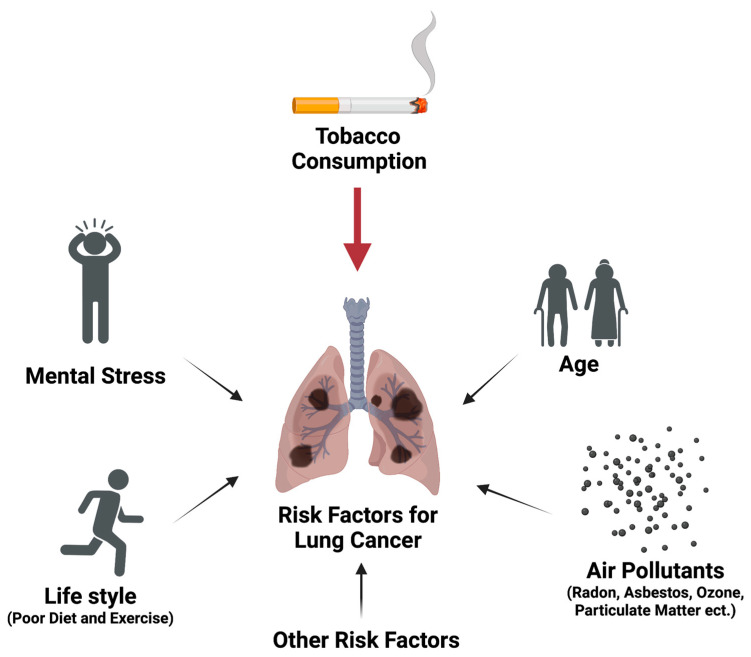
Risk factors for lung cancer development.

Like most chronic diseases, lung carcinogenesis is a slow and complicated process that includes many risk factors (Figure 1). The most well-known and proven risk factor for lung cancer is tobacco exposure. Tobacco consumption has been associated with the development of many types of cancers [23]. In a 30-year follow-up study, both male and female smokers were observed to have a higher overall cancer incidence rate in comparison to non-smokers [24]. The differences were more distinct when comparing lung cancer incidence between smokers and non-smokers (2.96% male smokers vs. 0.22% male non-smokers and 2.31% female smokers vs. 0.15% female non-smokers) [24]. Tobacco use, mainly through cigarette smoking, has been estimated to contribute to 80–90% of lung cancer causes [25]. Indeed, lung cancer has progressed from a rare disease to the leading cause of cancer mortality worldwide due to the widespread consumption of tobacco products in recent decades [26]. Tobacco-related diseases, specifically lung cancer, remain a leading cause of mortality worldwide and have put a significant strain on global health systems [27,28]. Tobacco carcinogens can be classified into multiple chemical classes. The two major classes are polycyclic aromatic hydrocarbons (PAHs) and *N*-nitrosamines. Others include aromatic amines, aldehydes, volatile organic hydrocarbons, and metals. Cigarette smoke also contains free radicals from reactive oxygen and nitrogen species that contribute to lung cancer risk through oxidative stress [29].

Beyond tobacco exposure, many other risk factors have been implicated to induce or facilitate lung carcinogenesis, including age, genetics, lifestyle, and exposure to pollutants and certain chemicals such as air pollutants, asbestos, radon, nickel, cadmium, benzidine, vinyl chloride, benzene, ozone, and particulate matter [30]. Radon, a radioactive gas produced through the decay of uranium-238, is considered the largest risk factor following tobacco exposure [31]. The dominant source for radon exposure comes from indoor accumulation whereby the radon gas can leak through foundations and concentrate to hazardous levels [32]. In a pooling study comparing residential radon exposure and lung cancer incidence, participants exposed to ≥ 200 Bq/m^3^ of radon gas had an odds ratio of 1.73 (95% CI: 1.27–2.35) to develop lung cancer [33].

In addition to these risk factors, there has been a recent push to understand the potential role of mental stress on lung cancer incidence. Mental stress can comprise multiple factors such as acute and chronic life events, work stress, personality, coping style, and depression [34,35]. In a study investigating the role of mental stress on cancer incidence and survival using meta-analysis, high stress-related psychosocial factors were associated with a higher lung cancer incidence with a hazard ratio of 1.23 [36]. There have also been studies investigating cancer incidence as it relates to a patient’s stress resilience. In a cohort study, lower stress resilience in men was associated with a higher risk of lung cancer with a hazard ratio of 2.75 (95% CI: 2.02–3.74) [37]. While these studies suggested the potential contribution of mental stress to lung cancer risk, more work is needed to improve study design and eliminate potential biases to define its quantitative contribution. One potential complementary strategy could be the objective measurement of the biological mediators of the stress pathways, such as stress hormones in different biospecimens.

These different risk factors either cause or facilitate lung carcinogenesis via multiple mechanisms. One main mechanism by which these risk factors lead to lung tumor initiation is to induce DNA damage (formation of modified DNA or DNA adducts) in the target lung tissues. Many carcinogenic compounds in tobacco and other sources undergo biotransformation into reactive species, dominantly through cytochrome P450 enzymes (CYP)-mediated oxidation or hydroxylation. For example, PAHs are typically metabolized by CYPs to form diol epoxides as the reactive species, capable of forming a covalent bond with DNA through a cis or trans addition of the exocyclic amino groups of purines to diol epoxides [38]. In a mechanism similar to PAHs, N-nitrosamines such as nicotine-derived nitrosamine ketone (NNK) undergo CYP-mediated α-hydroxylation to generate a reactive diazonium cation, which also forms a covalent bond with a DNA base or the phosphate backbone [39]. These DNA adducts, if not repaired, have the potential to introduce genetic mutations and trigger lung carcinogenesis. In addition to modifying DNA through covalent bond formation with carcinogens, tobacco and air pollutants also induce DNA damage through oxidative stress. Tobacco smoke has been shown to induce oxidative damage to DNA through increased production of reactive oxygen species (ROS) [40]. NNK has also been implicated as one of the compounds found in tobacco, responsible for oxidative DNA damage through the formation of 8-hydroxyguanine (8-OH-Gua) [41]. Radon decay also causes oxidative stress through the production of alpha particles from ionizing radiation [42]. The radiation can produce ROS species and damage DNA [42], which could lead to mutagenicity and thus carcinogenesis.

Tobacco, radon, chronic stress, and other risk factors have also been proposed to initiate or facilitate lung tumorigenesis via DNA damage independent mechanisms. The main reported mechanisms include the stimulation of inflammatory pathways or the suppression of immune functions. For instance, PAHs have been shown to be immunosuppressive through the alteration of antigen and mitogen receptor signaling and activation of apoptotic genes in immune cells [43]. NNK has been shown to stimulate inflammation and suppress immune functions. Specifically, NNK could upregulate the expression of cyclooxygenase 2 (COX-2) [44], a key enzyme in prostaglandin biosynthesis and an inflammatory signal mediator. An increase in prostaglandin synthesis, specifically prostaglandin-E2 (PGE2), can upregulate IL-10 and other inflammatory mediators. Upon upregulation, IL-10 can suppress cytotoxic activity of alveolar macrophages (AM) and natural killer cells (NK) through inhibition of IL-12 [45]. Cigarette smoke and radon may also stimulate inflammation through the production of ROS species, which can lead to impairment of epithelial and endothelial cellular function [46]. The damaged cells have an increased likelihood of becoming mutated. Chronic stress has been shown to dysregulate the hypothalamic–pituitary–adrenocortical axis (HPA), which can lead to increased cortisol levels as well as glucocorticoid receptor resistance (GCR) [47]. GCR can desensitize immune cells and prolong the release of proinflammatory cytokines [48].

Many other factors may also contribute to increased lung cancer risks, such as diet, sleep, and physical activities through diverse mechanisms. Furthermore, these different risk factors may contribute differentially to the lung cancer risk depending on individual heterogeneity. For instance, although tobacco smoke contributes to 80–90% of the cause of lung cancer, only a small fraction of smokers eventually develops lung cancer in their lifetime. Non-tobacco risk factors may have varied contributions to lung cancer risk among these individuals. At the same time, most of these risks likely contribute cumulatively to lung carcinogenesis over an extended period of time. Irrespective of etiologies, strategies should be implemented to target every step of these processes to help effectively reduce lung cancer risks and improve lung cancer outcomes.

## 3. Lung Tumorigenesis Animal Models, Advantages, and Limitations, Particularly with Respect to Physiological Relevance for Clinical Translation

With multiple risk factors and multiple mechanisms involved in human primary lung carcinogenesis, the lung carcinogenesis model used to evaluate the preventive candidates/strategies, ideally, needs to recapitulate these carcinogenic features so that the results will have a higher relevance for human translation. Given that carcinogen bioactivation and the consequent DNA damage has been proposed as the root cause of lung carcinogenesis, biochemical- or cell-based models are not widely used or accepted in evaluating and developing lung cancer preventive agents due to their limited and compromised metabolic capacity. Therefore, a range of animal models have been developed and utilized to evaluate lung cancer preventive agents. However, these animal models have their own strengths and weaknesses, and should be taken into consideration for their selection and data interpretation, particularly for those studies aiming for human translation. The same preventive candidates have been reported to reveal different efficacies in preventing lung carcinogenesis among different animal models, emphasizing the importance of considering their potential relevance for human translation. Although there are four major subtypes of lung cancers, no single lung tumorigenesis animal model to date can capture all of the pathological subtypes of human lung cancer [49,50,51,52]. Several animal models relevant to kava’s lung cancer preventive potential will be discussed herein with their advantages and limitations.

### 3.1. High-Dose Tobacco Carcinogen (NNK or BaP)-Induced Lung Tumorigenesis A/J Mouse Models

Lung tumors can be induced in A/J mice with a single dose or several doses of NNK and/or BaP. These animal models have been widely used because of several key advantages—the simple procedures for lung tumor induction (a single dose or several dosages of carcinogens via i.p. injection or gavage), the high and reproducible tumor load, and the relatively short period for lung tumor formation (17–26 weeks). The aforementioned features make them an economical system to screen for lead candidates (less effort, smaller number of animals needed to have enough statistical power, and shorter experimental periods). Therefore, the high-dose lung tumorigenesis models have been widely used in the early discovery stage of lung cancer preventive agents, including our own efforts that have demonstrated the potential of kava in lung cancer prevention [53,54]. However, these models have significant limitations for human translation based on our own experience. Firstly, the dosages of the carcinogens used acutely (~50–100 mg/kg bodyweight) are hundreds and thousands of times higher than the levels of typical human exposure. The high-dose exposure likely activates pathways and processes that have compromised or no physiological relevance in humans. Indeed, we discovered that some metabolic processes and carcinogenesis observed at a high-dose NNK regimen were not detectable when the dose of NNK was reduced to levels more comparable to human exposure (manuscript in preparation). Second, the nature of low-dose chronic tobacco exposure observed in humans, chronic and potentially mild oncogenic signaling, including inflammation, and associated pathological changes are not captured in these acute high-dose carcinogen animal models. For instance, the increase in COX-2 in the single-dose NNK carcinogen model was observed to be transient right after carcinogen exposure with no signs of pathological lung inflammation detected (unpublished data). Such chronic oncogenic signaling, inflammation, and associated pathological changes are potentially physiologically critical in human lung cancer development and thus essential to be recapitulated in the animal model for prevention translational evaluation. The acute high-dose tobacco carcinogen-induced lung tumorigenesis animal models therefore are probably not sufficient to evaluate lung cancer preventive agents. This is a particularly important consideration for future human translation of candidates with multiple pharmacologic mechanisms.

### 3.2. Chronic Low-Dose NNK in Drinking Water Induced Lung Tumorigenesis Model

Lung tumors can also be induced in A/J mice upon a 7-week NNK exposure in drinking water. In comparison to the high-dose models, this model captures chronic lung inflammation, which is probably why a number of anti-inflammatory agents demonstrated measurable lung cancer preventive efficacy in this model [55,56,57] but not in the high-dose carcinogen models [55]. Hecht et al. recently showed that F344 rats upon chronic low-dose NNK exposure in drinking water (5 ppm over one year) formed ADC and adenosquamous carcinoma [58]. More importantly, some lung tumors were aggressive, resulting in pancreatic metastasis [59]. The mechanisms of the underlying carcinogenesis and metastasis, in addition to carcinogen bioactivation and DNA damage, remains to be determined. Similarly, Schuller et al. reported that hamsters upon frequent NNK exposure (twice weekly at 12.5 mg/kg) developed adenosquamous lung tumors [60]. The results suggest that a chronic low-dose NNK treatment regimen, better mimicking its exposure in human smokers, may be a critical factor for lung tumor formation, both in pathology and invasion/metastasis. The low-dose NNK models, however, still have several limitations. First, only a single chemical from tobacco smoke, NNK, was used to induce lung tumor formation, which does not capture the risks conferred by other tobacco ingredients, such as PAHs. Second, NNK is administered in drinking water or i.p. injection; thus its lung exposure is expected to be substantially different from NNK exposure in the form of tobacco smoke, which may likely bypass liver metabolism.

### 3.3. A Chronic Tobacco Smoke-Exposure Induced Lung Tumorigenesis A/J Mouse Model

The chronic tobacco smoke exposure-induced lung tumorigenesis A/J mouse model is probably the most physiologically relevant lung carcinogenesis animal model reported to date. This model was initially introduced in the decades following World War I as the United States aimed to find a concrete connection between tobacco smoking and lung cancer incidence. The model lost its popularity by the 1970s due to repeated failures in multiple animal species [61]. In the 1990s, an interest in the dangers of tobacco smoke as well as chemoprevention, among others, revived this model. Initially, this model involved 24-h tobacco smoke exposure 7 days a week, often with inconclusive results. The 24-h continuous tobacco smoke exposure probably does not mimic the temporal tobacco exposure among human smokers as well. In the early 2000s, Witschi et al. introduced a different tobacco exposure regimen: 5 months of tobacco smoke exposure at 6 h a day, 5 days a week, followed by a 4-month recovery period [62]. Across 11 independent studies of this style, analysis showed increased lung tumor incidence compared to the control mice exposed to filtered air, as well as an overall positive correlation between tobacco smoke exposure and tumor multiplicities [62]. Other studies of similar design have shown comparable results, suggesting that the recovery period is key to tumor development. It remains to be determined whether the 6-h daily exposure is optimal, which is still substantially longer than the daily tobacco exposure among human smokers. A shorter daily tobacco exposure that is more representative among smokers, for instance 2 h a day, may be able to promote lung tumorigenesis without the needed recovery period, which should be tested in the future. While this model is time and labor intensive, requiring special facilities and appropriate training for personnel, it is potentially a more accurate model of human lung cancer. Additionally, other health conditions correlated with tobacco smoke can be assessed as well, such as chronic obstructive pulmonary disease (COPD) [63] and heart functions [64]. This opens the opportunity to understanding the potential interactions of these diseases on the incidence and progression of lung cancer, which have not yet been systematically evaluated. Because of its high cost, long experimental period, and relatively low tumor load in the tobacco exposure group, this model has not been used in the discovery phase of lung cancer preventive agents. However, it has been employed in the translational phase of phenethyl isothiocyanate (PEITC), epigallocatechin gallate (EGCG), green tea, myo-inositol, and dexamethasone [65,66,67,68]. The results from this model have provided strong justification for their clinical evaluations. As discussed, this model has several key advantages despite its high cost and long experimental period. First, the exposure regimen (5-month tobacco exposure and 4-month recovery) mimics tobacco exposure and lung cancer development among active smokers and former smokers, who typically start tobacco use in early adulthood and have years of tobacco exposure before quitting. Second, such a physiologically relevant model offers the opportunity to evaluate the preventive potential of candidate agents with the administration covering varied periods relative to tobacco exposure, mimicking current and former smokers. Third, such a model allows the holistic evaluation of the candidate agents, particularly for the potential impacts on other complications caused by tobacco smoke and the long-term safety. Given the limited success of developing lung cancer preventive agents to date using the simplified animal models, the smoke exposure animal model should be reconsidered, particularly for potential candidates before human clinical translation.

At the same time, the models discussed above only include tobacco smoke as the risk factor for lung cancer. To further enhance human relevance, additional risk factors should be integrated in the lung carcinogenesis animal models, such as stress, diet, air pollution, and physical activities. Certain simple stress paradigms have been built on the high-dose carcinogen models and have been demonstrated to enhance or accelerate the lung carcinogenesis process. Stress paradigm models have been used to evaluate lung cancer preventive candidates targeting the mental stress pathways, such as β-adrenergic receptor antagonists. One limitation is that the molecular and pathological changes have not been well characterized in such models. Another limitation is the level of stress being applied has not been quantified or estimated in comparison to the stress levels among humans—the level of stress from the applied stress paradigms, such as physical constraints, may well exceed the typical levels of chronic stress human smokers experience. In addition, such a stress paradigm condition has not been integrated with a tobacco smoke-induced lung carcinogenesis animal model to potentially better mimic human smokers.

There are additional lung tumorigenesis animal models, including some that use non-tobacco carcinogens or genetically engineered mouse models (GEMM). These models have different levels of human physiological relevance. The GEMM models may be amendable to evaluate the interception effect on different subtypes of lung malignancies. Details of their advantages and limitations can be found in other review articles [69,70]. Among different GEMMs, lung tumors formed are mostly ADCs. Recently, GEMMs with LKB1 mutational loss of function have been reported to form different subtypes of lung cancers [71,72,73,74,75,76,77]. Specifically, mice with LKB1 loss of function in conjunction with K-ras [75], PTEN [76] or SOX2 [77] developed metastatic lung tumors of varied pathologies, including ADC, SCC, and large-cell carcinoma. Reduction in LKB1 copy number was also observed in the early stage of human lung cancers, indicating that its loss of function may play a role in human primary lung tumorigenesis [78]. Moreover, LKB1 deficiency sensitizes mice to carcinogen (DMBA)-induced lung tumorigenesis in the form of SCC [79]. In our recent studies, NNK and its metabolite NNAL have been found to deactivate LKB1 via phosphorylation both in vitro and in vivo [80], which may contribute to NNK-induced primary lung carcinogenesis independent of inducing DNA damage. Studies are ongoing to characterize the contribution of such a LKB1 loss of function to NNK-induced lung tumorigenesis.

## 4. Kava’s Potential, Mechanisms, and Challenges in Cancer Risk Reduction, Particularly Lung Cancer

### 4.1. Knowledge about Kava, Its Traditional Use and Potential Benefits

**Figure 2:**
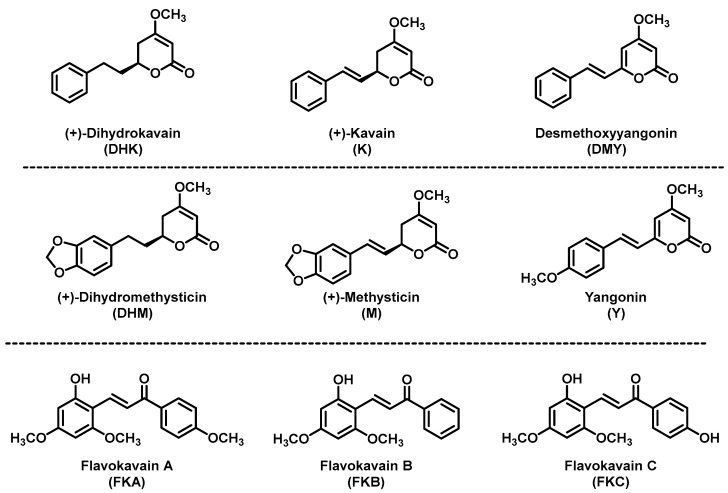
Structures of the six major kavalactones and the three flavokavains in kava.

Traditionally, kava is a beverage prepared from the root of *Piper methysticum*, which belongs to the pepper family, that originated and is dominantly cultivated in the South Pacific islands. Kava has been consumed by indigenous peoples for centuries as a religious and celebratory drink [81]. Kava gradually evolved into a common beverage among the South Pacific Islanders due to its relaxing properties [82,83,84,85]. The active components in kava for relaxation include lactone-based compounds termed kavalactones [86] with kavain, dihydrokavain, methysticin, dihydromethysticin, yangonin, and desmethoxyyangonin as the six major ones (Figure 2).

Despite their high structural similarity, these natural kavalactones have distinct pharmacokinetic [87] and pharmacodynamic properties [88,89]. It is therefore possible that these kavalactones may be complementary to each other and none of them individually will be able to fully recapitulate the holistic beneficial properties of kava. Other than kavalactones, a class of chalcone based compounds have been detected in kava products and heavily investigated, named flavokavains A, B, and C (Figure 2) [90]. These flavokavains have not been reported to contribute to kava’s relaxing property. A number of putative targets have been reported for kavalactones, including voltage-gated sodium and calcium ion channels, gamma-aminobutyric acid (GABA) type A receptors, and monoamine oxidase B (MAO-B), with detailed information in our previous review [91].

Historically, the types of kava products (chemotype) have been characterized by their relative abundance of individual kavalactones [92]. Kava products of different chemotypes have been proposed to possess varied benefits and risks, because of different composition profiles of kavalactones and flavokavains [93]. Other than in the format of a drink, kava has also been commercialized in the form of capsules or tinctures as dietary supplements. Because of these variables, currently available kava products could be very diverse due to their difference in format, kavalactone abundance and profiles, and the content of other ingredients, such as flavokavains A and B [94,95,96]. Not surprisingly, various benefits and risks could potentially be introduced due to these chemical composition variations. Commercial kava products thus should be rigorously standardized with accurate content information for human use, particularly in the case of potential chronic use, such as its potential use in primary lung carcinogenesis prevention [95].

### 4.2. Epidemiological Data Supporting Kava in Cancer Risk Reduction

In the year 2000, Steiner first proposed the potential of kava to reduce human cancer risk [97]. Briefly, an inverse relationship between kava consumption and cancer incidence among several islands in the South Pacific was reported by Steiner, leading to the hypothesis that kava may have the potential to reduce cancer risk (Table 1).

In addition, cancer incidence rates were lower in males relative to females in South Pacific nations with higher kava consumption, which is the opposite of global trends, as previously mentioned [97]. Given that traditional kava is dominantly consumed by males, the lower cancer incidence among males versus females in nations with high kava consumption also supports kava’s potential to prevent cancer. In this report, cancer incidence includes all types of cancers. It is possible that kava may have differential effects among different cancer types, which has not yet been rigorously investigated. A limited number of potential confounding variables other than kava were briefly analyzed as well, such as smoking rate, which was found to be comparable among those nations and may not contribute to the observed cancer incidence differences [98]. Several other epidemiological data also indicate lower cancer incidence among males in comparison to females in the South Pacific [99,100], which is again opposite to the global trend [1], further substantiating kava’s potential in reducing human cancer risk.

### 4.3. Kava’s Potential in Cancer Risk Reduction in Animal Models, Responsible Ingredients, and Mechanisms

Stimulated by these interesting human epidemiological data, kava’s potential to reduce cancer risk has been evaluated during the past two decades using various chemical-induced or transgenic animal models, including lung, prostate, colon, and bladder tumorigenesis [53,54,89,101,102,103,104,105,106,107,108,109,110,111]. In these animal models, tumorigenesis was induced by genetic mutations or different chemical carcinogens via different administration routes. Kava, via gavage or in the form of diet, has also been administered via different regimens, either during or after carcinogen exposure in the chemical carcinogenesis model. The positive results of kava to prevent tumorigenesis in all of these animal models strongly suggest that kava may reduce human cancer risk, likely via different mechanisms. In fact, different kavalactones have been identified as the active ingredients in some of these carcinogenesis models. For instance, dihydromethysticin has been identified as one active compound that can effectively suppress NNK-induced lung carcinogenesis in A/J mice [89] while kavain was recently identified to prevent bladder carcinogenesis induced by hydroxy butyl(butyl) nitrosamine (OH-BBN) in mice [111]. Although kavain has not been evaluated for its potential against NNK-induced lung carcinogenesis, it is less likely to be as effective as dihydromethysticin in this model based on its lack of efficacy in reducing NNK-induced DNA damage in target lung tissue [89]. These results also argue that maybe none of the single-chemical entities in kava are capable of fully recapitulating the holistic benefits of kava in cancer risk reduction, upon which the human epidemiological data are built. This is particularly important for human translation. Kava, a natural blend of kavalactones with historical human exposure and epidemiological support, may be the ideal candidate instead of any single chemical from kava as long as the kava product has rigorous quality control and quality assurance.

With respect to its potential in preventing lung carcinogenesis, kava was first evaluated against lung carcinogenesis induced by eight oral dosages of NNK and BaP in A/J mice [53]. Kava was supplemented in the diet with three different treatment regimens, covering only the carcinogen exposure period (mimicking current smokers), covering the postcarcinogen exposure period (mimicking former smokers), and covering the whole experimental period. In all of these treatment regimens, kava significantly reduced the number of lung tumors, indicating kava’s potential to reduce lung cancer risk among both current and former smokers [53]. Preliminary mechanistic investigation suggests that kava inhibited the activation of nuclear factor kappa B (NF-κB) [53]. Given that chronic lung inflammation is a well-established risk factor for lung cancer, kava may prevent lung carcinogenesis in this animal model at least in part by suppressing tobacco-induced lung inflammation. Chalcone-based flavokavains (Figure 2) in kava were initially hypothesized as the responsible active ingredients since many chalcone-based compounds have been reported with cancer preventive potential in various animal models [112]. Our data later rejected this hypothesis as flavokavains from kava, at several dosages, failed to capture the preventive efficacy of kava in this animal model [104]. Additional medicinal chemistry efforts from our lab were able to develop analogs of the flavokavains with a wide range of bioactivity in cell models, but none of them were able to block lung carcinogenesis in this animal model and some compounds showed significant toxicity (unpublished data). The traditional approach for active ingredient identification, fractionation and biological evaluation, was adopted to search for the active chemicals [54]. The fraction enriched with kavalactones was able to recapitulate the preventive efficacy of kava in a two-dose NNK-induced lung carcinogenesis A/J mouse model and dihydromethysticin was identified as an active compound [54]. Dihydrokavain was demonstrated completely inactive in this animal model, which later was used as a control compound for mechanistic elucidation. Based on their distinct effects in reducing NNK-induced DNA damage, methysticin is likely active as well while kavain would not in this animal model [54]. Extensive structure–activity relationship studies have been performed on dihydromethysticin to characterize the functional groups important for its lung cancer preventive activity [101,102,108] but to date none of the synthetic compounds were able to outperform the natural dihydromethysticin except the unnatural enantiomer of dihydromethysticin. It should be noted that the dose range of kava and natural dihydromethysticin, with effective lung cancer prevention in these animal studies, was comparable to the levels of traditional kava consumption in humans. Thus, in alignment with the epidemiologic data, kava may be potent enough to reduce human lung cancer risk in its natural format.

**Figure 3:**
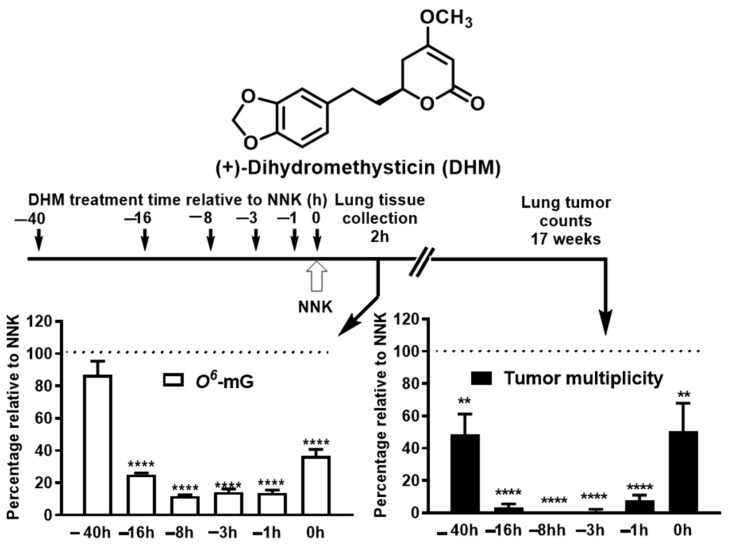
Temporal effects of DHM on NNK-induced lung DNA damage and tumors (data reformatted from references). One-way ANOVA to compare means among NNK and NNK + DHM timepoints. Dunnett test to compare the means between NNK and a specific NNK + DHM timepoint. **: *p* < 0.01; ****: *p* < 0.0001.

In the two-dose NNK-induced lung tumorigenesis model, one mechanism of kava to prevent lung carcinogenesis is to enhance NNK detoxification and thus reduce NNK-induced DNA damage [106,107]. The enhanced NNK detoxification is likely mediated via the transcriptional upregulation of UDP-Glucuronosyltransferase (UGT) enzymes, resulting in increased glucuronidation and urinary excretion of NNAL [102,106,107], which may offer the potential of precision prevention given the genetic variations in UGTs in humans. At the same time, we observed that kava and dihydromethysticin may prevent lung carcinogenesis via DNA damage independent mechanisms in this animal model. Using dihydromethysticin as the example, complete tumor blockage was achieved with an incomplete protection against DNA damage (i.e., 75–88% reductions, Figure 3). Furthermore, dihydromethysticin given 40 h before NNK exposure reduced tumor multiplicity by 52% with little DNA damage reduction, while dihydromethysticin given concurrent with NNK reduced tumor multiplicity by only 50% although DNA damage was reduced by 63% [102]. Therefore, dihydromethysticin achieves complete prevention against NNK-induced lung carcinogenesis likely via both DNA damage-driven and -independent mechanisms [102]. The DNA damage-independent carcinogenic mechanism of NNK, however, had not been rigorously elucidated in this animal model until our recent study [44].

At the same time, chronic lung inflammation is a well-established risk factor for lung carcinogenesis. Several kavalactones have demonstrated anti-inflammatory activities in vivo [85,113,114,115,116,117,118,119,120,121,122]. For instance, kavain inhibits lipopolysaccharide (LPS)-induced collagen antibody induced arthritis in mice [120]. Desmethoxyyangonin inhibits LPS-induced inflammation and LPS/D-galactosamine-induced hepatitis in mice [113]. Therefore, kava may be able to reduce lung cancer risk partly through its anti-inflammatory activities. However, the two-dose NNK-induced lung tumorigenesis animal model, as discussed above, does not appear to recapitulate the chronic inflammatory nature of lung cancer risk in humans. Future work is needed to characterize the anti-inflammatory contribution of kava to reduce lung cancer risk via clinically more relevant animal models. Kava may also reduce lung cancer risk through its relaxing property if chronic mental stress is a valid risk for lung cancer. Thus, the potential contribution of stress reduction to kava’s lung cancer risk also requires future investigation. Indeed, kava revealed the potential to reduce tobacco use and tobacco dependence among smokers in a pilot clinical trial [123], which may be mediated through its relaxing properties as reflected by the reduction in the plasma levels of cortisol [123]. In summary, kava may reduce lung cancer risk via multiple mechanisms, namely reducing tobacco use and dependence, enhancing tobacco carcinogen detoxification and thus reducing DNA damage, suppressing tobacco smoke-induced lung inflammation, and promoting relaxation. How to holistically evaluate these potential benefits in a physiologically relevant animal model remains to be a major challenge.

With respect to the underlying molecular signaling for stress reduction and inflammation suppression, we found that NNK in tobacco smoke and its metabolite NNAL may function as β-adrenergic receptor agonists and modulate the PKA/LKB1/CREB/COX-2 pathway in A/J mouse lungs; kava and dihydromethysticin effectively suppressed the effects of NNAL on this pathway [44]. Specifically, we performed an RNA seq analysis of the A/J mouse lung tissues from control, NNK, and NNK + dihydromethysticin, respectively. Genes significantly modified by NNK relative to control were identified (3282 genes, *p* < 0.05). Similarly, genes significantly modified by dihydromethysticin relative to NNK were identified (1886 genes, *p* < 0.05). A total of 984 genes were found in common in both comparisons (*p* = 5.27 × 10^−225^). Importantly, 89.3% of them were modified by NNK and DHM in opposite directions. These results indicate that dihydromethysticin counteracts the signaling processes induced by NNK. These genes were subjected to the Ingenuity Pathway Analysis (IPA). Protein Kinase A (PKA) was predicted as one of the top signaling pathways activated by NNK but suppressed by dihydromethysticin [44]. The classical PKA pathway has been well characterized: stress hormones, such as norepinephrine, bind to and activate β-adrenergic receptor (β-AR). This activates adenylyl cyclase for cAMP production. cAMP then binds to the regulatory subunit of PKA, consequently releasing and activating PRKACA (the catalytic subunit). Activated PRKACA phosphorylates CREB and induces CREB-mediated transcription, which results in the up-regulation of COX-2 that may contribute to tobacco smoke-induced lung inflammation. NNK indeed has been reported by Schuller et al. as a potent agonist for β-AR, through which it can promote NSCLC proliferation [124]. Activated PRKACA also phosphorylates LKB1, rendering LKB1 loss of its tumor suppressive function [80]. This signaling pathway was further confirmed via multiple cell models using a tobacco carcinogen metabolite, NNAL, at physiologically relevant concentrations [44,80]. Norepinephrine, the stress hormone, appears to be able to modulate the same signaling pathway (unpublished results), which may be the underlying mechanism of stress as a risk factor to primary lung carcinogenesis. In addition, these signaling events have been well-documented to contribute to cancer development and progression, including lung cancer. First, PKA activation alone has been demonstrated to be sufficient to drive primary tumorigenesis [125,126,127] in several lab animal models, including lung cancer. Indeed, PKA was identified as the key driver oncoprotein via un-biased global profiling in multiple studies [126,127]. Second, the systemic levels of PRKACA have been observed to be elevated in patients of various types of cancers, including lung cancer, and thus PRKACA in blood has been proposed as a potential cancer biomarker [128,129,130,131,132]. Third, PKA activation has been reported to induce anxiety in multiple animal models as well [133,134,135,136] while kava is well-known for its anxiolytic (anti-anxiety) property [91]. Finally, the oncogenic and inflammatory functions of PKA, CREB, LKB1 loss of function and COX-2 in lung tumorigenesis have been well documented [126,137,138,139]. These results overall suggest that the β-adrenergic receptor-mediated PKA/LKB1/CREB/COX-2 signaling pathway could be one main mechanism in promoting lung carcinogenesis with tobacco smoke and mental stress as the potential stimuli.

Although the focus of this review is kava’s potential in primary lung cancer prevention, compounds in kava, primarily flavokavains, have also been reported to reveal anticancer potentials. Specifically, flavokavains A and B have been reported to exhibit anticancer activity in multiple cancer cell models including lung, breast [140], bladder [141], and prostate [142]. They were able to inhibit cancer cell proliferation [143], angiogenesis [144], metastasis [145], or modulate immune responses [146,147].

### 4.4. Potential Risks Associated with Kava Use, Particularly in the Chronic Use

Besides these potential benefits, kava has been associated with hepatotoxic potential even though the risk was rated extremely rare and idiosyncratic with a wide range of mechanisms and responsible ingredients proposed, including chalcone-based flavokavains and various kavalactones [148]. Specifically, chalcone-based flavokavains A and B have been demonstrated to induce hepatotoxicity in animal models or potentiate acetaminophen (APAP)-induced hepatotoxicity, potentially via the depletion or reduction in endogenous glutathione level [149]. The content of flavokavains A and B indeed were found to be higher in kava plants not recommended for human use in comparison to those popular for traditional consumption. Kavalactones have also been suspected to contribute to kava’s hepatotoxic risk, potentially through drug herb interactions since various kavalactones have been reported to modulate several CYP enzymes [150,151]. The concentrations or doses of kavalactones evaluated in the biochemical or cell-based models may have limited human physiological relevance based on our recent pharmacokinetic studies of kava [87]. Nonetheless, the safety of kava needs to be closely monitored in future human translation, particularly for its potential chronic use in lung cancer risk reduction.

## 5. Strategies and Opportunities for Future Kava Translational Development in Reducing Lung Cancer Risk

### 5.1. Rationale to Evaluate Kava Instead of Any Single-Chemical Entity in Kava

As discussed, lung cancer is induced by multiple risk factors. Thus, a single-chemical entity is less likely to achieve effective lung cancer prevention, due to the low probability of it exhibiting polypharmacological effects against different risk factors. Indeed, this is well demonstrated in the past lung cancer preventive endeavors between individual chemicals vs. natural mixtures, such as green tea vs. EGCG and vegetable juice vs. PEITC or indole-3-carbinol. Specifically, EGCG, PEITC, and indole-3-carbinol were identified as the active ingredients from green tea and vegetables via cell-based or simplified animal models, but all of them failed to fully recapitulate the efficacy of their corresponding natural mixture entities in later translation. Similarly, our data and the numerous literature reports suggest that despite the structural similarities of the different compounds present in kava, such as kavalactones, they each have distinct beneficial activities. They may act complementarily to each other to achieve the polypharmacological goal for effective lung cancer risk reduction, such as carcinogen detoxification, inflammation inhibition, and stress reduction in addition to their distinct pharmacokinetic properties. Furthermore, kava’s potential to reduce cancer risk is based on kava’s traditional use by humans in the form of its natural mixture rather than any single-chemical entities (Table 2). Lastly and potentially most importantly, the historical long-term human consumption data provide a key safety foundation for the translation of natural blend kava, which is currently lacking for any of its single-chemical entities.

### 5.2. Evaluating Kava’s Preventive Potential in a Clinically More Relevant Lung Carcinogenesis Animal Models and Developing Mechanism-Based Non-Invasive Clinically Translatable Biomarkers

As discussed earlier, the chronic tobacco smoke-induced lung tumorigenesis A/J mouse model is probably the most clinically relevant lung carcinogenesis animal model despite its high cost and comparatively low number of tumors per mouse, which requires a larger number of animals for adequate sample size. This model has key advantages. (1) It captures the chronic nature of several key risk factors, including tobacco exposure, lung inflammation, and stress potentially due to nicotine addiction. (2) In addition, the tobacco exposure regimen in this model nicely mimics current and former smokers, who typically start tobacco use in early adulthood for decades followed by quitting. (3) It offers the opportunity to evaluate the preventive potential of candidate agents covering varied periods relative to tobacco exposure, mimicking prevention among current and former smokers. (4) The chronic tobacco exposure nature of this model also offers the opportunity to evaluate the holistic effects of kava on general health conditions, including risks of non-lung cancer diseases imposed by chronic tobacco exposure, such as COPD and cardiovascular diseases, although these have not been systematically characterized in this animal model in previous studies. Given the great promise of kava as a lung cancer preventive agent, we propose that it is well justified to evaluate and confirm the lung cancer preventive efficacy of kava in this most physiologically relevant animal model and simultaneously explore kava’s potential impact on other tobacco smoke-related health conditions. Equally important, mechanism-based clinically translatable biomarkers need to be discovered and developed using this animal model. Such non-invasive biomarkers are instrumentational to facilitate future clinical translation. The chronic kava consumption in this model also offers the opportunity to evaluate the chronic safety profile of kava, which is again essential to future clinical translation. At the same time, further improvement in animal models may be needed, such as the integration of other lung cancer risk factors, to better mimic lung carcinogenesis in humans.

### 5.3. Identification and Intervention for Individuals with Higher Lung Cancer Risk and Timely Efficacy Monitoring for Precision Prevention and Interception

As discussed earlier, effective prevention of lung cancer has been challenging, partly because (1) tobacco control and tobacco cessation have not been very successful; (2) due to addiction, many smokers are willing to accept the lung cancer risk associated with tobacco use as the majority of them will not develop lung cancer clinically; and (3) the challenges to timely monitor the efficacy of lung cancer prevention and to ensure the safety of the chronic use of preventive agents. It is therefore important to identify and intervene with high-risk individuals who are in more urgent need for lung cancer prevention. Moreover, the efficacy of any lung cancer preventive agent is expected to be heterogenous among participants such that some may benefit more than others. It is critical to identify the individuals who will likely benefit the most from preventive intervention. Such individuals could be identified or at least enriched upon obtaining a detailed understanding of the mechanisms underlying lung carcinogenesis and kava-based lung cancer prevention through the integration of animal studies and pilot clinical trials. Based on the current understanding of kava-based lung cancer prevention, one particular genetic opportunity is UGTs, which are involved in the detoxification of tobacco carcinogens, including NNK. Many UGTs have genetic variants in humans, some of which have been reported to be associated with differential lung cancer risks among smokers. On the basis of our preclinical and pilot clinical studies, kava treatment increases the glucuronidation of NNAL and its urinary detoxification, contributing to its lung cancer preventive activity. It is important to identify the responsible UGTs, which may provide guidance in identifying the populations more likely to benefit from the enhanced detoxification introduced by kava. Similar principles may be applicable to other genetic factors involved in lung cancer risk and kava’s preventive activities, such as CYP enzymes which are critical for the bioactivation of various tobacco carcinogens. Kava has been reported to modulate the activities of various CYP enzymes ex vivo although the concentrations used may not be physiologically relevant. Additional research is needed to explore whether kava treatment in humans indeed modulates the CYP enzymes and if so, whether such CYPs may be involved in lung cancer risks. Chronic stress is another potential risk factor for lung cancer while kava is well known for its anti-stress properties and thus may reduce lung cancer risk through stress reduction. Genetic factors involved in the β-AR mediated PKA/LKB1/CREB/COX-2 pathway thus may influence kava’s efficacy in reducing lung cancer risk, which remains to be investigated. In addition, mechanism-based non-invasive clinically translatable biomarkers are essential to improve the feasibility of clinical translation of kava in lung cancer prevention. Upon developing a panel of clinically translatable biomarkers capturing kava’s carcinogen detoxification mechanisms [158,159,160], we were able to evaluate the potential of kava to reduce lung cancer risk among smokers via a pilot clinical trial with one-week kava consumption [123]. Such biomarkers also have the potential to identify the population more likely to benefit from kava use. The timely monitoring also offers the potential to tailor the treatment regimen to maximize kava’s benefits and minimize its risk if any. Besides its potential to prevent primary lung carcinogenesis, kava also has the potential to prevent/intercept the progression of fully transformed lung cancer (unpublished data) and to improve the quality of life among lung cancer survivors with the potential to delay cancer recurrence.

### 5.4. Rigorous Quality Control and Quality Assurance of Kava Product

The rigor quality control and quality assurance of the kava products is essential for animal evaluation and human translation. As discussed earlier, there are a wide range of kava products with many factors that influence its composition and in turn, its pharmacology and toxicology. Upon deeper understanding of the mechanism of kava in lung cancer prevention and identifying the responsible ingredients, a kava product of well-defined chemical composition with rigorous quality control and quality assurance is essential for its development and implementation in lung cancer prevention.

### 5.5. Safety of Chronic Kava Use

Lastly and importantly, the safety of chronic kava use in humans needs to be closely monitored in its clinical translation, particularly given the tremendous heterogeneity in humans, including genetic, environmental, and other factors, that may have different levels of tolerance to kava exposure. The compliance and acceptance of kava use among participants are also critical for its potential as a lung cancer preventive agent given the necessity of its chronic use.

## 6. Summary

**Figure 4:**
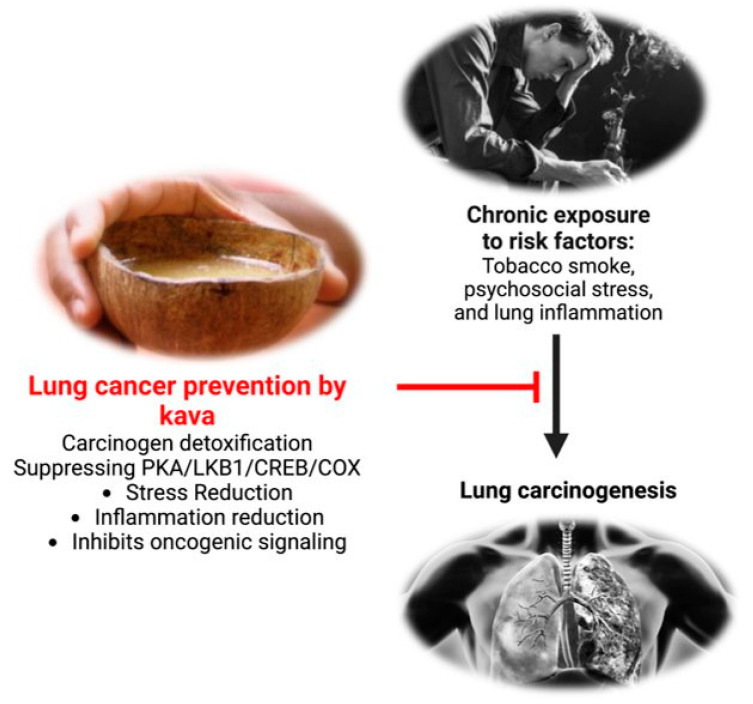
Potential points of lung carcinogenesis prevention through the use of kava.

In summary, lung cancer prevention should be an important pillar for its effective management. This review has documented a promising potential for kava and its phytochemicals in reducing human lung cancer risk, supported by its epidemiological observations, preventive efficacy of lung carcinogenesis in multiple animal models, associated mechanisms, and the mechanism-based biomarker changes in pilot human trials. Its polypharmacological effects via multiple mechanisms potentially induced from multiple components could be essential to practical lung cancer risk reduction in humans (Figure 4). To facilitate eventual clinical translation, current animal models need further optimization with better human physiological relevance, capturing multiple lung cancer risk factors. Genetic and mechanistic knowledge of kava in lung cancer risk reduction is also critical to help identify the high-risk individuals more likely to benefit from kava intervention. Mechanism-based noninvasive clinically translatable biomarkers are needed as well to help identify the target population and to timely monitor the intervention efficacy for precision intervention regimen. Upon addressing these challenges via the integration of animal models and pilot clinical trials, kava’s potential in reducing human lung cancer risk will be more rigorously characterized and quantified in more adequately powered clinical evaluations, which will pave the way for its future translation and implementation.

## Figures and Tables

**Table 1:** The cancer incidence in males and females and the estimated amount of kava consumed in different nations (reformatted from [97]).

Age-Standardized Cancer Incidence Rates for All Sites per 100,000 Population in 1960s–1970s			
Country	Male	Female	Kava Consumed/Person/Year (Kilograms)
Vanuatu	70.9	83.7	6.7
Fiji	75	112.2	2.8
Western Samoa	90.2	93.7	2.2
Micronesia	132.9	97	1.4
New Caledonia	182	154	0.6
Hawaii	311.9	297.6	0
New Zealand	322.9	297.6	0
USA, Los Angeles	307.2	276.2	0

**Table 2:** Different biological activities of compounds in kava and related references.

Activity of Compounds in Kava and Related References								
	DHM	M	Y	DHK	K	DMY	FKA	FKB
Carcinogen detoxification and DNA damage reduction in mice [89]	++	+	-	-	-	-	-	-
PK in human [87] and mice [152]	++	+	-	++	+	-	N/A	N/A
NE-induced cAMP in cells [153]	+	N/A	++	-	-	+	N/A	N/A
Anxiolytic activity in human [154] ^#^	N/A	N/A	N/A	N/A	+	N/A	N/A	N/A
Anxiolytic activity in chicken [152,155] ^#^	N/A	N/A	N/A	+	N/A	N/A	N/A	N/A
Anti-inflammatory activity in mice [85,113,114,115,116,117,118,119,120,121,122] ^#^	+	+	+	N/A	+	+	N/A	N/A
Hepatotoxic risk in mice [156,157]	-	-	-	-	-	-	+	+

^#^: not all kavalactone compounds were evaluated at the same time so their efficacies cannot be compared. The most active candidate thus remains to be determined. N/A: not available. ++: strong activity; +: moderate activity; -: no activity.

## Data Availability

No new data were created.

## Abbreviations

8-OH-Gua	8-hydroxyguanine
ADC	Adenocarcinoma
AM	Alveolar macrophage
APAP	Potentiate acetaminophen
β-AR	β-Adrenergic receptor
cAMP	Cyclic adenosine monophosphate
COPD	Chronic obstructive pulmonary disease
COX-2	Cyclooxygenase-2
CTC	Circulating tumor cell
CtDNA	Cell free circulating tumor DNA
CYP	Cytochrome P450 enzyme
DHK	Dihydrokavain
DHM	Dihydromethysticin
DMY	Desmethoxyyangonin
EGCG	Epigallocatechin gallate
FKA	Flavokavain A
FKB	Flavokavain B
GCR	Glucocorticoid receptor resistance
GEMM	Genetically engineered mouse models
HPA	Hypothalamic-pituitary-adrenocortical axis
i.p.	Intraperitoneal
IPA	Ingenuity pathway analysis
LCC	Large cell carcinoma
LDCT	Low-dose CT
LPS	Lipopolysaccharide
NF-κB	Nuclear factor kappa B
NK	Natural killer cell
NLST	The National Lung Screening Trial
NNK	Nicotine-derived nitrosamine ketone
NSCLC	Non-small-cell lung cancer
OH-BBN	Hydroxy butyl(butyl) nitrosamine
PAH	Polycyclic aromatic hydrocarbons
PEITC	Phenethyl isothiocyanate
PGE2	Prostaglandin-E2
PKA	Protein kinase A
ROS	Reactive oxygen species
SCC	Squamous cell carcinoma
SCLC	Small cell lung cancer
SOX2	SRY-box 2
TEP	Tumor-educated platelet
UGT	UDP-Glucuronosyltransferases
